# Postoperative delirium is associated with grey matter brain volume loss

**DOI:** 10.1093/braincomms/fcad013

**Published:** 2023-01-30

**Authors:** Ilse M J Kant, Jeroen de Bresser, Simone J T van Montfort, Theodoor D Witkamp, Bob Walraad, Claudia D Spies, Jeroen Hendrikse, Edwin van Dellen, Arjen J C Slooter, BioCog consortium, Claudia Spies, Georg Winterer, Tobias Pischon, Diana Boraschi, Reinhard Schneider, Peter Nürnberg, Malte Pietzsch Norman Zacharias, Rudolf Morgeli, Maria Olbert, Gunnar Lachmann, Friedrich Borchers, Kwaku Ofosu, Fatima Yurek, Alissa Wolf, Jurgen Gallinat, Jeroen Hendrikse, Arjen Slooter, Edwin van Dellen, Emmanuel Stamatakis, Jacobus Preller, David Menon, Laura Moreno-Lopez, Stefan Winzeck, Insa Feinkohl, Paola Italiani, Daniela Melillo, Giacomo Della Camera, Roland Krause, Karsten Heidtke, Simone Kuhn, Marion Kronabel, Thomas Bernd Dscietzig, Franz Paul Armbruster, Bettina Hafen, Jana Ruppert, Axel Bocher, Anja Helmschrodt, Marius Weyer, Katarina Hartmann, Ina Diehl, Simon Weber, Ariane Fillmer, Bernd Ittermann

**Affiliations:** Department of Intensive Care Medicine and University Medical Center Utrecht Brain Center, University Medical Center Utrecht, Utrecht University, Utrecht 3584 CX, The Netherlands; Department of Information Technology and Digital Innovation, Leiden University Medical Center, Leiden 2333 ZA, The Netherlands; Department of Radiology, Leiden University Medical Center, Leiden 2333 ZA, The Netherlands; Department of Intensive Care Medicine and University Medical Center Utrecht Brain Center, University Medical Center Utrecht, Utrecht University, Utrecht 3584 CX, The Netherlands; Department of Radiology and University Medical Center Utrecht Brain Center, University Medical Center Utrecht, Utrecht University, Utrecht 3584 CX, The Netherlands; Department of Intensive Care Medicine and University Medical Center Utrecht Brain Center, University Medical Center Utrecht, Utrecht University, Utrecht 3584 CX, The Netherlands; Department of Anesthesiology and Operative Intensive Care Medicine (CCM, CVK), Charité – Universitätsmedizin Berlin, Berlin 10117, Germany; Department of Radiology and University Medical Center Utrecht Brain Center, University Medical Center Utrecht, Utrecht University, Utrecht 3584 CX, The Netherlands; Department of Intensive Care Medicine and University Medical Center Utrecht Brain Center, University Medical Center Utrecht, Utrecht University, Utrecht 3584 CX, The Netherlands; Department of Psychiatry and University Medical Center Utrecht Brain Center, University Medical Center Utrecht, Utrecht University, Utrecht 3584 CX, The Netherlands; Department of Intensive Care Medicine and University Medical Center Utrecht Brain Center, University Medical Center Utrecht, Utrecht University, Utrecht 3584 CX, The Netherlands; Department of Psychiatry and University Medical Center Utrecht Brain Center, University Medical Center Utrecht, Utrecht University, Utrecht 3584 CX, The Netherlands; Department of Neurology, UZ Brussel and Vrije Universiteit Brussel, Jette 1090, Belgium

**Keywords:** delirium, encephalopathy, brain volume, brain infarcts, grey matter

## Abstract

Delirium is associated with long-term cognitive dysfunction and with increased brain atrophy. However, it is unclear whether these problems result from or predisposes to delirium. We aimed to investigate preoperative to postoperative brain changes, as well as the role of delirium in these changes over time. We investigated the effects of surgery and postoperative delirium with brain MRIs made before and 3 months after major elective surgery in 299 elderly patients, and an MRI with a 3 months follow-up MRI in 48 non-surgical control participants. To study the effects of surgery and delirium, we compared brain volumes, white matter hyperintensities and brain infarcts between baseline and follow-up MRIs, using multiple regression analyses adjusting for possible confounders. Within the patients group, 37 persons (12%) developed postoperative delirium. Surgical patients showed a greater decrease in grey matter volume than non-surgical control participants [linear regression: B (95% confidence interval) = −0.65% of intracranial volume (−1.01 to −0.29, *P* < 0.005)]. Within the surgery group, delirium was associated with a greater decrease in grey matter volume [B (95% confidence interval): −0.44% of intracranial volume (−0.82 to −0.06, *P* = 0.02)]. Furthermore, within the patients, delirium was associated with a non-significantly increased risk of a new postoperative brain infarct [logistic regression: odds ratio (95% confidence interval): 2.8 (0.7–11.1), *P* = 0.14]. Our study was the first to investigate the association between delirium and preoperative to postoperative brain volume changes, suggesting that delirium is associated with increased progression of grey matter volume loss.

## Teaser

Kant *et al*. report that patients with postoperative delirium have an increased progression of grey matter loss compared to patients without postoperative delirium. These changes may contribute to impaired long-term cognitive outcomes of postoperative delirium.

## Graphical Abstract

**Graphical abstract:**
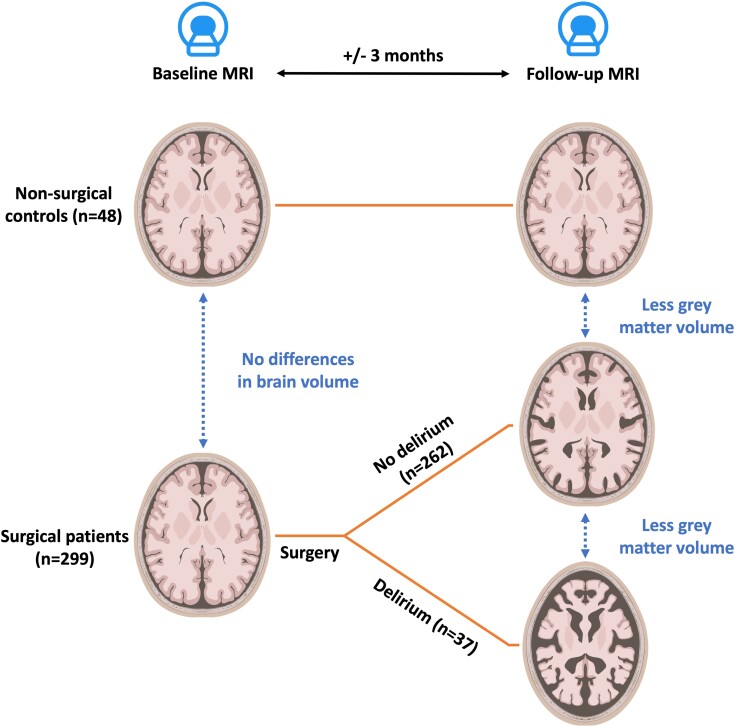

## Introduction

Delirium, a clinical expression of an acute encephalopathy,^1^ is characterized by an acute change in attention, awareness and other cognitive disturbances.^2^ It is common in elderly patients, particularly after major surgery.^2^ Delirium is a risk factor for long-term cognitive deficits, such as mild cognitive impairment and dementia.^3,4^ However, pre-existing cognitive deficits are also a major risk factor for delirium.^2^ It is therefore unclear whether the association of delirium with long-term cognitive impairment is causal.

A number of neuroimaging studies have linked delirium to several forms of brain changes, which, among others, include atrophy white matter hyperintensities (WWH) and brain infarcts.^5–7^ For example, a small study in cardiac surgery patients showed that postoperative delirium was associated with new brain infarcts in the immediate postoperative period.^8^ Furthermore, longitudinal diffusion MRI suggested that postoperative delirium is associated with decreased fractional anisotropy in the frontal, temporal and parietal regions, which might reflect damaged white matter.^9^

However, previous longitudinal studies that assessed the relation between delirium and brain changes over time are limited, lacked (complete) baseline imaging data^10–12^ and were underpowered.^8^ Therefore, it remains a matter of debate whether delirium and the underlying encephalopathy result in additional brain changes, or whether patients who develop delirium are already on a trajectory of brain deterioration. Investigating the relation between delirium and longitudinal brain changes with imaging before the onset of delirium can improve our understanding of delirium and long-term adverse outcomes.

The aim of this study was to investigate preoperative to postoperative brain changes, as well as the role of delirium in these changes over time. The brain changes that were assessed included brain volumes, WWH and brain infarcts.

## Materials and methods

### Study design and participants

This study was part of an observational longitudinal multicentre study that aims to identify biomarkers for postoperative delirium and postoperative cognitive dysfunction: the ‘Biomarker Development for Postoperative Cognitive Impairment in the Elderly’ (BioCog) study, that has been described in detail elsewhere.^13^ Patients were included in two participating centres: Charité Universitätsmedizin Berlin (Berlin, Germany, centre 1), and University Medical Center Utrecht (Utrecht, The Netherlands, centre 2). Control participants were recruited from general practitioner’s offices in Berlin, Germany and Utrecht, The Netherlands, matched on age and sex on a group level. Inclusion criteria for patients and control participants were: (i) ≥65 years of age; (ii) a mini-mental state exam (MMSE) score of ≥24, as this may reflect preoperative cognitive impairment which could interfere with the results and (iii) ability to undergo MRI scanning and cognitive testing. The patient group was scheduled for major surgery of ≥60 min, the control group was not scheduled for surgery in the upcoming 12 months.

### Standard protocol approvals, registrations and patient consent

The study protocol was approved by the medical ethical committees of both centres under ethical approval number EA2/092/14 (centre 1) and 14/469 (centre 2). All participants signed written informed consent. The BioCog study was registered at clinicaltrials.gov identifier NCT02265263.

### Procedures

Patients and non-surgical control participants were invited for a baseline visit (patients: before surgery), which included a brain MRI scan, MMSE and clinical assessments on medical history and vascular risk factors. Trained researchers administered the MMSE and questionnaires. The preoperative American Society of Anesthesiologists (ASA) score for patients was scored by an anaesthesiologist (in training). Three months after surgery (patients), and 3 months after baseline examinations (non-surgical controls), all participants were invited for a follow-up visit which included a second brain MRI scan and questionnaires.

### Delirium assessment

Delirium was defined according to the fifth edition of the diagnostic and statistical manual of mental disorders criteria.^14^ Following surgery, trained researchers performed a delirium assessment twice daily until the seventh postoperative day or until discharge, whichever came first, using the confusion assessment method for the intensive care unit (CAM-ICU)^15^ and the nursing Delirium screening scale (Nu-DESC).^16^ In addition, a validated chart review^17^ was performed daily to assess additional signs of delirium. Delirium assessments were performed until the seventh postoperative day, as delirium afterwards is unlikely to be related to anaesthesia and surgery. Patients were considered delirious in case of a positive CAM-ICU score and/or ≥2 cumulative points on the Nu-DESC and/or patient chart review that showed descriptions of delirium (e.g. confused, agitated, drowsy, disorientated, delirious and receiving antipsychotic therapy because of delirium). Duration of delirium was defined as the cumulative number of days that a patient was delirious according to these criteria.

### Brain MRI scans

Participants were scanned on a 3 T Magnetom TrioTim (Siemens Healthcare, Erlangen, Germany) MRI scanner (centre 1) or a 3 T Achieva (Philips Healthcare, Best, The Netherlands) (centre 2). The MRI scanning protocol was standardized between both centres and consisted of a three-dimensional (3D) T_1_-weighted sequence [voxel size 1.0 × 1.0 × 1.0 mm^3^; centre 1: repetition time (TR)/echo time (TE) 2500/4.77 ms; centre 2: TR/TE 7.9/4.5 ms] a fluid-attenuated inversion recovery (FLAIR) sequence (centre 1: TR/TE/inversion time 4800/388/1800 ms; voxel size 0.49 × 0.49 × 1.00 mm^3^; centre 2: TR/TE/inversion time 4800/125/1650 ms; voxel size 1.11 × 1.11 × 0.56 mm^3^) and a diffusion-weighted image [centre 1: n.a.; centre 2: (voxel size = 0.96 × 1.19 × 4.00 mm^3^; TR/TE 3294/68 ms)] for visual inspection only.

### MRI processing steps and analysis

The MRI processing method that was used is relatively robust for scanner differences^18^ and has previously been described in another BioCog substudy.^19^ All processing steps were performed using statistical parametric mapping version 12 (Wellcome Institute of Neurology, University College London, UK, http://www.fil.ion.ucl.ac.uk/spm/doc/) for Matlab (The MathWorks, Inc., Natick, Massachusetts, USA). In short, 3D FLAIR images were registered to 3D T_1_-weighted images. White matter hyperintensity probability maps were calculated on the registered 3D FLAIR images using the lesion segmentation toolbox (version 2.0.15, www.statistical-modeling.de/lst.html) and the lesion prediction algorithm. A lesion-filling method from the lesion segmentation toolbox was performed on the T_1_-weighted images. The filled T_1_-weighted images were segmented in grey matter, white matter and cerebrospinal fluid, and intracranial volume (ICV) were estimated by the computational anatomy toolbox (CAT12), version r1155.^20^ All segmentations were visually checked by a trained researcher (I.K.), supervised by a neuroradiologist (J.B.). All brain volumes were expressed as a percentage of the ICV of that time point, for example, baseline grey matter volumes are shown as a percentage of the baseline ICV (relative brain volume). Differences in brain volumes (delta brain volume) between time points are shown as the crude difference between the relative brain volumes of baseline and follow-up measurements. Cerebral infarcts were scored by two neuroradiologists (T.W. and J.B.) who were blinded to the patient characteristics, by use of the T_1_-weighted, FLAIR and diffusion weighted images.

### Statistical analysis

Socio-demographics of patients and control participants were compared with an independent-samples *t*-test for continuous, normally distributed data, with a χ^2^ for categorical data, and with a Mann–Whitney-*U-*test for continuous, skewed data.

Changes in brain volume or white matter hyperintensity volume (delta volume) between study groups were assessed with separate linear regression analyses with volume change (delta volume) as dependent variable. All linear regression analyses were adjusted for age, sex, time between baseline and follow-up measurements, centre, baseline brain volume and vascular risk factors: diabetes, body-mass index (BMI), smoking, hypertension, hyperlipidaemia and history of transient ischaemic attack (TIA)/stroke. For example, in grey matter volume, the analysis was adjusted for baseline grey matter volume. Analysis of changes within patient groups was additionally adjusted for the type of surgery. The association between delirium and new postoperative cerebral infarcts was studied with logistic regression analysis with new brain infarcts as the dependent variable, and adjustments for age, sex, time between baseline and follow-up measurements and centre.

In secondary analyses, brain volume changes in patients with delirium were compared to the control participants, and brain volume changes in patients without delirium were compared to control participants by linear regression analyses that were adjusted for the same possible confounders. Further, we performed a sensitivity analysis on brain volume changes, which excluded all patients with an MMSE score below 28.

Further, to assess whether the occurrence of new brain infarcts could explain an association between postoperative delirium and a change in grey matter volume, a mediation analysis was performed, according to the method by Preacher and Hayes.^21^ Hence, the analysis of delirium with preoperative to postoperative change in grey matter volume was adjusted for new brain infarcts, in addition to all previously mentioned possible confounders. The difference and relative change in the effect estimates of postoperative delirium with the preoperative to postoperative change in grey matter volume were studied.

All statistical analyses were performed in IBM SPSS Statistics version 25. A *P*-value < 0.05 was considered statistically significant.

## Results

### Patients and control participants: characteristics

In total, 299 patients and 48 control participants were included in this study, who all had a baseline and follow-up MRI scan (see Supplementary Fig. 1 for a flowchart on the reasons for in- and exclusion). As shown in Table 1, patients and control participants showed no differences in the distribution of age and sex. Patients more often had hypertension. Median time between measurements in the patients was longer than in the controls [128 (interquartile range 98–147) days versus 108 (interquartile range 91–133) days, *P* = 0.02].

**Table 1:** Demographics of the total group of patients and non-surgical control participants

	Surgical patients (*n* = 299)	Non-surgical controls (*n* = 48)	*P*-value
Age	72 (5)	71 (5)	0.76
Sex (female)	101 (34%)	21 (44%)	0.20
Study centre			<0.001
Centre 1	155 (52%)	8 (17%)	
Centre 2	144 (48%)	40 (83%)	
Baseline MMSE	29 (28–30)	29 (28–30)	0.58
Vascular factors			
Hypertension	175 (59%)	17 (35%)	0.003
Hyperlipidaemia	102 (34%)	13 (33%)^a^	0.89
BMI	27 (24–29)	26 (24–29)	0.19
Diabetes	52 (17%)	8 (17%)	1.00
Current smoker	32 (11%)	6 (13%)	0.81
History of TIA/stroke	28 (9%)	5 (13%)^a^	0.57

Data represent *n* (percentage), mean (SD) or median (interquartile range). An independent-samples *t*-test was performed on continuous data, and an Mann–Whitney *U*-test for non-normal distributed data. A χ^2^ comparison of two groups was performed for categorical data. MMSE: mini-mental state exam.

a % missing hyperlipidaemia and history of TIA/stroke in, respectively, patients and controls: hyperlipidaemia *n* = 3 (1%), *n* = 9 (19%); TIA/stroke: *n* = 3 (1%), *n* = 8 (17%).

### Patients with and without postoperative delirium: characteristics

Within the patient group, 37 persons (12%) developed postoperative delirium during the first 7 days after surgery. The median duration of delirium was 2.5 (interquartile range 1–4) days. Patients with postoperative delirium were generally older, were more often admitted to the intensive care unit (ICU), had a higher ASA score and were more often scheduled for cardiac surgery than patients without postoperative delirium (Table 2). No differences were found in the median follow-up time between the preoperative and postoperative MRI scan between patients with and without postoperative delirium [delirium: 116 (interquartile range 99–162) days; no delirium: 114 (interquartile range 97–147) days, *P* = 0.36]. Patients with postoperative delirium had a longer median duration of hospitalization [delirium: nine (interquartile range 7–14) days, no delirium: four (interquartile range 2–7) days, *P* < 0.01], and were more often admitted to the ICU [delirium: 46% (*n* = 17), no delirium: 16% (*n* = 41)].

**Table 2:** Demographics of patients with postoperative delirium and without postoperative delirium

	Postoperative delirium (*n* = 37)	No postoperative delirium (*n* = 262)	*P*-value
Age	73 (3)	71 (5)	0.04
Sex (female)	14 (38%)	87 (33%)	0.58
Study centre			0.49
Centre 1	17 (46%)	138 (53%)	
Centre 2	20 (54%)	124 (47%)	
ICU admission (≥1 day)	15 (41%)	32 (12%)	<0.001
Preoperative MMSE	28 (27–30)	29 (28–30)	0.09
Preoperative ASA			0.02
I	2 (5%)	21 (8%)	
II	19 (51%)	171 (65%)	
III	16 (43%)	70 (27%)	
Type of surgery			0.01
Cardiac	10 (27%)	24 (9%)	
Gastro-intestinal/abdominal	14 (38%)	85 (33%)	
Orthopedic	7 (19%)	87 (34%)	
Other*	6 (16%)	66 (25%)	
Vascular factors			
Hypertension	22 (60%)	153 (59%)	1.00
Hyperlipidaemia	15 (41%)	87 (34%)	0.46
BMI	26 (23–28)	27 (24–29)	0.16
Diabetes	8 (22%)	44 (17%)	0.49
Current smoker	3 (8%)	29 (11%)	0.82
History of TIA/stroke	7 (19%)	21 (8%)	0.06

Data represent *n* (percentage), mean (SD) or median (interquartile range). An independent samples *t*-test was performed on continuous data, and an Mann–Whitney *U*-test for non-normal distributed data. A χ^2^ comparison of two groups was performed for categorical data. *Other types of surgery: plastic, breast, ear nose throat, endocrine and maxillofacial. MMSE: mini-mental state exam. ASA: classification of disease severity for the American Society of Anesthesiologists. BMI: body-mass index. ICU: intensive care unit. TIA: transient ischaemic attack.

Postoperative delirium occurred in 16% (*n* = 84) of the total group of patients who were included in the BioCog study and who had a baseline MRI scan, including patients without follow-up MRI. Among the patients who came back for a follow-up MRI and thus were included in the current study, delirium occurred in 12% (*n* = 37), indicating a greater loss to follow-up in the group with postoperative delirium (Supplementary Fig. 1).

### Preoperative to postoperative brain changes in patients and control participants

Compared to control participants, patients showed a larger decrease over time in total brain volume [B_adj_ = −0.50% ICV, 95% confidence interval (CI) −0.83 to −0.18, *P* < 0.01], and grey matter volume [B_adj_ = −0.65% ICV (95% CI −1.01 to −0.29), *P* < 0.01] (Table 3). Further, compared to control participants, patients did not show a larger change over time in white matter volume or white matter hyperintensity volume (Table 3). There was further a non-significantly increased risk of a new brain infarct during follow-up between patients [6% (*n* = 16)] and controls [3% (*n* = 1)], odds ratio (95% CI): 2.43% (0.26–22.53), *P* = 0.44.

**Table 3:** Preoperative to postoperative brain volume changes in patients and non-surgical controls

	Surgical patients			Non-surgical controls			B (95% CI)
	Preoperative	Postoperative	Δ	Preoperative	Postoperative	Δ	
Total brain	72.1 (2.8)	71.8 (2.7)	−0.3 (0.9)	72.0 (3.2)	72.2 (3.2)	0.2 (0.8)	−0.50 (−0.83–−0.18)*
Gray matter	39.5 (2.0)	39.3 (2.0)	−0.2 (0.1)	39.7 (2.0)	40.0 (2.0)	0.2 (0.9)	−0.65 (−1.01–−0.29)**
White matter	32.6 (2.0)	32.5 (2.0)	−0.1 (0.7)	32.3 (2.0)	32.2 (2.2)	0.0 (0.6)	0.16 (−0.09–0.41)
Cerebrospinal fluid	27.9 (2.7)	28.2 (2.7)	0.3 (0.8)	28.0 (3.2)	27.8 (3.2)	−0.2 (0.8)	
WMH	0.17 (0.07–0.36)	0.18 (0.08–0.39)	0.02 (0.08)	0.34 (0.07–0.58)	0.35 (0.08–0.40)	0.02 (0.08)	0.02 (−0.01–0.05)

Crude volumes are shown as the percentage of the ICV of that time point as mean (standard deviation) or median (interquartile range). B coefficients of the difference in volume change over time between patients and control participants (in % ICV) are shown with a 95% CI and were adjusted for age, sex, study centre, ICV, time between measurements and baseline volume (for example, in gray matter, the B was adjusted for preoperative gray matter volume). Crude cerebrospinal fluid volumes were shown, but not analysed as total brain volume was analysed. WMH: white matter hyperintensities. Number of included patients and control participants per analysis: brain volumes patients: *n* = 262, control participants: *n* = 42. White matter hyperintensities patients: *n* = 246, control participants: *n* = 44. *P = 0.003. **P < 0.0005.

### Preoperative to postoperative brain changes in patients with and without postoperative delirium

Delirium was associated with a greater decrease in preoperative to postoperative grey matter volume [B_adj_ = −0.44% ICV (95% CI −0.82 to −0.06), *P* = 0.02] (Table 4). Further, delirium duration was associated with a greater decrease in preoperative to postoperative grey matter volume [B_adj_ = −0.20% (−0.38 to −0.02), *P* = 0.03]. White matter volume and white matter hyperintensity volume showed no difference over time between patients with and without postoperative delirium (Table 4). Patients with postoperative delirium further showed a higher occurrence of new brain infarcts in the postoperative period than patients without postoperative delirium, although this did not reach statistical significance [delirium: 12% (*n* = 5), no delirium: 4% (*n* = 11) odds ratio (95% CI): 2.8 (0.7–11.1), *P* = 0.14].

**Table 4:** The association between postoperative delirium and preoperative to postoperative brain volume changes

	Postoperative delirium			No postoperative delirium			B (95% CI)
	Preoperative	Postoperative	Δ	Preoperative	Postoperative	Δ	
Total brain	71.9 (2.8)	71.3 (2.9)	−0.5 (1.0)	72.1 (2.8)	71.8 (2.7)	−0.3 (0.8)	−0.29 (−0.64–0.05)
Gray matter	39.5 (2.1)	39.0 (2.0)	−0.5 (1.1)	39.5 (2.0)	39.3 (1.9)	−0.2 (0.9)	−0.44 (−0.82 – −0.06)*
White matter	32.3 (1.9)	32.3 (1.8)	0.0 (1.0)	32.6 (2.0)	32.5 (2.0)	−0.1 (0.7)	0.15 (−0.11–0.42)
Cerebrospinal fluid	28.1 (2.8)	28.6 (2.9)	0.5 (1.0)	27.9 (2.7)	28.2 (2.6)	0.3 (0.8)	
WMH	0.14 (0.05–0.42)	0.19 (0.06–0.42)	0.02 (0.10)	0.17 (0.08–0.36)	0.18 (0.08–0.39)	0.02 (0.08)	−0.02 (−0.05–0.02)

Crude volume changes are shown as the delta percentage of the ICV of that time point, mean (standard deviation) or median (interquartile range). B coefficients of the difference in volume change over time between patients with and without delirium are shown with a 95% CI and were adjusted for age, sex, study centre, ICV, type of surgery, time between measurements and baseline volume (for example, in gray matter, the beta was adjusted for preoperative gray matter volume). ICV: intracranial volume. WMH: white matter hyperintensity. Crude cerebrospinal fluid volumes were shown, but not analysed as total brain volume was analysed. Number of included patients and control participants per analysis: brain volumes, delirium: *n* = 28, no delirium: *n* = 234. White matter hyperintensities, delirium: *n* = 26, no delirium: *n* = 220. *P = 0.02.

Delirium patients had a greater decrease over time in total brain volume and grey matter volume than control participants [total brain volume: B_adj_ = −0.73% ICV (95% CI −1.24 to –0.22), *P* < 0.01; grey matter volume: B_adj_ = −0.81% ICV (95% CI −1.38 to −0.24), *P* < 0.01]. Patients without postoperative delirium also showed a larger decrease over time in total brain volume and grey matter volume than control participants [total brain volume: B_adj_ = −0.41% ICV (95% CI −0.70 to −0.13), *P* < 0.01; grey matter volume: B_adj_ = −0.38% ICV (95% CI −0.69 to −0.07), *P* = 0.02].

A sensitivity analysis on the association between delirium and preoperative to postoperative grey matter volume change in which patients with an MMSE score below 28 were excluded (delirium group: *n* = 17, no delirium group: *n* = 196), showed no association between delirium and pre- to postoperative grey matter volume change [B_adj_ = −0.16% ICV (95% CI −0.60 to 0.28), *P* = 0.48].

### Mediation analysis

The association between postoperative delirium and preoperative to postoperative change in grey matter volume (result: B1) was additionally adjusted for new brain infarcts (result: B2). The difference of these estimates [B2—B1= −0.04 (95% CI −0.24 to 0.10)] did not differ from 0, indicating that new brain infarcts were not a mediator in the association of delirium with grey matter volume loss (see Supplementary Fig. 2 for all mediation coefficients).

## Discussion

In summary, we found that surgical patients showed a greater decrease in grey matter volume than non-surgical control participants. Within the patients group, postoperative delirium was associated with a greater decrease in grey matter volume.

We found that 12% of surgical patients developed postoperative delirium. This frequency is lower than observed previously (20–50%),^22^ but the latter findings come from older studies probably using older and more toxic analgesia regimes without delirium prevention programmes.^23,24^ In addition, we restricted to relatively cognitively healthy elective surgical candidates (i.e. baseline MMSE score ≥ 24), which likely further decreased the frequency of postoperative delirium.

### Surgery and brain volume loss

Brain volume loss over time is a feature of normal ageing.^25^ However, accelerated brain volume loss is one of the key features of cognitive impairment and dementia.^26^ To study brain volume loss, it is, therefore, crucial to follow a control group of the same age. Our results indicate that surgical patients showed more progression of cerebral atrophy in comparison with control participants. These findings may be explained by a higher disease burden in the patients group. Alternatively, this could be due to the consequences of anaesthesia and surgery, which might lead to an immune response with brain volume loss.^27,28^ Other forms of potential perioperative brain injury such as metabolic insufficiency (e.g. impaired oxygen delivery due to hypoxia, hypotension and/or microcirculatory changes), cerebral (micro-)emboli in case of cardiac surgery, and possibly changes in neurotransmitter activity may also alter brain structure and function.^2,22^ One may hypothesize that pre to postoperative brain volume loss might be due to the fact that patients were (over) hydrated preoperatively. However, in almost all patients, the preoperative MRI was performed when the patients were not yet admitted, so this explanation seems unlikely. Other explanations may be medication effects or postoperative complications. Interestingly, the group of patients without delirium still showed greater atrophy progression compared to the control participants, indicating that this effect was not only driven by delirium.

### Delirium and brain volume loss

Delirium is consistently associated with dementia, although the nature of this relationship remains poorly understood.^29^ It has been hypothesized that one of the contributing factors to long-term cognitive deficits after delirium could be an increased loss of brain volume.^10,11,30^ Two previous investigations in critically ill patients studied delirium and brain volumes and showed that delirium was associated with a lower brain volume at ICU discharge and 3 months thereafter,^11^ and that ICU admission may be associated with accelerated brain volume loss, particularly in patients who experienced delirium.^12^ Another study that performed brain MRI after cardiac surgery showed that delirium was associated with a lower postoperative brain volume.^10^ However, these studies either lacked baseline imaging^5,6^ or had a limited number of baseline imaging assessments available,^12^ and could therefore not conclude whether brain atrophy would be cause or consequence of delirium.

Using MRI scans made before the onset of delirium, we observed accelerated brain volume loss in patients who were delirious, which was supported by a statistically significant dose-response relation between duration of delirium and grey matter volume loss. A rate of 0.44% loss of brain volume as observed in this study, corresponds to a total brain volume loss of −1.76% within one year, or a 4-fold increased rate compared to cohorts of community-dwelling older adults.^31–35^

A possible explanation for the accelerated brain volume loss in patients who had postoperative delirium could be persisting neuroinflammation.^36^ In elderly people who are vulnerable to delirium, microglia might already be primed, which may lead to overactivation of new stimuli, such as systemic inflammation due to surgery. Overactivated microglia may then release cytotoxic substances that ultimately lead to neuronal damage, which may persist despite recovery of the underlying illness.^36^ Interestingly, a sensitivity analysis excluding patients with an MMSE score <28 showed that the effect was mostly driven by patients with a lower MMSE score. These patients could therefore be more prone to brain volume loss and might be at risk for further cognitive deterioration.

### Delirium and brain infarcts

New brain infarcts after major surgery may contribute to adverse neurocognitive outcomes.^8,37,38^ A large study in non-cardiac surgery patients (*n* = 1114), and a smaller study in cardiac surgery patients (*n* = 98) showed an association between silent new postoperative brain infarcts in the immediate postoperative period and postoperative delirium.^8,37^ Our results confirm these previous findings in a more heterogeneous group of patients, by showing an association between postoperative delirium and new cortical brain infarcts 4 months after surgery, although this did not reach statistical significance. In population studies on ageing in community-dwelling older individuals, 4–5% of the participants develop a new cerebral infarct within 12 months.^33,39,40^ We found that 12% of patients with postoperative delirium had a brain infarct in a time interval of approximately 4 months, which corresponds to an approximately eight times increased risk compared to the general population.

Most of the infarcts found in our study were neurologically silent infarcts, and we were, therefore, unable to determine the exact moment of infarction. It remains unclear whether these new infarcts resulted from surgery, or occurred later in the postoperative period. A new peri- or postoperative infarct could thus be one of the precipitating factors leading to delirium, or patients with delirium could be more prone to develop new cerebral infarcts in the postoperative period. The association between delirium and new postoperative infarcts indicates that these patients are at increased risk for further cognitive deterioration, as the presence of (silent) brain infarcts increases the risk of cognitive decline and dementia.^41^ Mediation analysis showed that the association of delirium with grey matter volume loss was not driven by the presence of new brain infarcts.

### Strengths and limitations

Our study was the first investigation in which brain MRIs were made before the occurrence of delirium, enabling us to rule out that smaller pre-existing brain volumes explained the association of delirium with brain atrophy. To the best of our knowledge, no other previous study has investigated this association. Another strength is that this was a two-centre study that included a large, heterogeneous group of older patients, which increases generalizability. Furthermore, we included a control group that did not undergo surgery. Brain volumes and white matter hyperintensity volumes were semi-automatically quantified using a pipeline that is robust for centre differences.

Limitations of this study include that we had a limited group of delirious patients, partly due to a larger loss of follow-up in patients that experienced postoperative delirium. This might have led to an underestimation of the observed effect, as delirious patients probably had a higher disease burden. The relatively low frequency of postoperative delirium compared to other studies,^4,42,43^ was found despite an extensive delirium screening protocol, and could, therefore, be the result of our high MMSE cut-off restriction inclusion criteria. Virtually all MRI studies in delirium, including this current study, have been performed in elective surgical patients with relatively healthy cognition, while the vast majority of delirium cases results from older, more frail patients. Therefore, these results may relate to a specific subgroup of delirium patients, not necessarily to delirium in general. Another limitation may be that the time between the measurements differed between the patient and control group. However, follow-up time was adjusted for in the statistical analyses. As with all observational studies, residual confounding (e.g. from perioperative risk factors) may have occurred. Hence, we could not causally relate postoperative delirium to the increased progression of grey matter volume loss.

### Conclusion

Our study was the first to investigate the association between delirium and preoperative to postoperative brain volume changes, suggesting that delirium is associated with increased progression of grey matter volume loss. These changes could be part of the underlying structural correlate of adverse long-term cognitive outcomes of postoperative delirium.

## Supplementary Material

fcad013_Supplementary_Data

## Data Availability

The datasets generated and analysed during the current study are not publicly available as this is a sub study of a still ongoing consortium study, but may be available from the corresponding author on reasonable request.

## Appendix I

The BioCog consortium consists of Claudia Spies, Georg Winterer, Tobias Pischon, Diana Boraschi, Reinhard Schneider, Peter Nürnberg, Malte Pietzsch Norman Zacharias, Rudolf Morgeli, Maria Olbert, Gunnar Lachmann, Friedrich Borchers, Kwaku Ofosu, Fatima Yurek, Alissa Wolf, Jurgen Gallinat, Jeroen Hendrikse, Arjen Slooter, Edwin van Dellen, Emmanuel Stamatakis, Jacobus Preller, David Menon, Laura Moreno-Lopez, Stefan Winzeck, Insa Feinkohl, Paola Italiani, Daniela Melillo, Giacomo Della Camera, Roland Krause, Karsten Heidtke, Simone Kuhn, Marion Kronabel, Thomas Bernd Dscietzig, Franz Paul Armbruster, Bettina Hafen, Jana Ruppert, Axel Bocher, Anja Helmschrodt, Marius Weyer, Katarina Hartmann, Ina Diehl, Simon Weber, Ariane Fillmer and Bernd Ittermann.

## Abbreviations

ASA =: American Society of Anaesthesiologists
BioCog =: Biomarker Development for Postoperative Cognitive Impairment in the Elderly
CAM-ICU =: confusion assessment method for the intensive care unit
CI =: confidence interval
FLAIR =: fluid-attenuated inversion recovery
ICU =: intensive care unit
ICV =: intracranial volume
MMSE =: mini-mental state exam
Nu-DESC =: nursing Delirium screening scale
TE =: echo time
TR =: repetition time
