# Supplementary material for: Postoperative delirium is associated with grey matter brain volume loss
Source: Brain Commun. 2023 Jan 30;5(1):fcad013. doi: 10.1093/braincomms/fcad013 (PMC9933897; doi:10.1093/braincomms/fcad013)
Supplement: fcad013_Supplementary_Data [file fcad013_supplementary_data.docx]

Supplementary material to: Postoperative delirium is associated with

grey matter brain volume loss

Ilse M. J. Kant, Jeroen de Bresser, Simone J. T. van Montfort, Theodoor D. Witkamp, Bob Walraad, Claudia D. Spies, Jeroen Hendrikse, Edwin van Dellen, and Arjen J. C. Slooter on behalf of the BioCog consortium

Supplementary figure 1: inclusion flowchart of patients and control participants from baseline preoperative MRI scan to follow-up MRI, with exclusion of scans unfit for analysis.


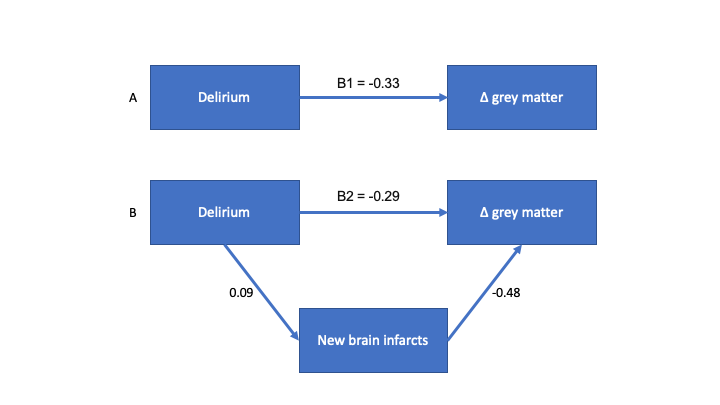


Supplementary figure 2 : mediation analysis of the association between delirium and grey matter volume loss. The association between postoperative delirium and preoperative to postoperative change in grey matter volume (A) was additionally adjusted for new brain infarcts (B). The difference of these estimates (B2 – B1= -0.04 (95% CI -0.24 to 0.10)) did not differ from 0, indicating that new brain infarcts were not a mediator in the association of delirium with grey matter volume loss (see Supplementary Figure 2 for all mediation coefficients).
